# Supplementary material for: Tuberculosis prevalence among children with severe acute malnutrition: a systematic review and meta-analysis
Source: medRxiv. 2026 Aug 14:2026.08.12.26360317. Preprint. [Version 1] doi: 10.64898/2026.08.12.26360317 (PMC13551367; doi:10.64898/2026.08.12.26360317)
Supplement: Supplementary file 1 [file media-1.pdf]

Table S1. PRISMA 2020 Checklist

Section & Topic	Item #	Checklist item	Location where item is reported
**TITLE**			
Title	1	Identify the report as a systematic review.	p. 1
**ABSTRACT**			
Abstract	2	See the PRISMA 2020 for Abstracts checklist.	p. 3
**INTRODUCTION**			
Rationale	3	Describe the rationale for the review in the context of existing knowledge.	p. 4 & 5
Objectives	4	Provide an explicit statement of the objective(s) or question(s) the review addresses.	p. 5
**METHODS**			
Eligibility criteria	5	Specify the inclusion and exclusion criteria for the review and how studies were grouped for the syntheses.	p. 5 & 6
Information sources	6	Specify all databases, registers, websites, organisations, reference lists and other sources searched or consulted to identify studies. Specify the date when each source was last searched or consulted.	p. 5
Search strategy	7	Present the full search strategies for all databases, registers and websites, including any filters and limits used.	Table S2
Selection process	8	Specify the methods used to decide whether a study met the inclusion criteria of the review, including how many reviewers screened each record and each report retrieved, whether they worked independently, and if applicable, details of automation tools used in the process.	p. 6
Data collection process	9	Specify the methods used to collect data from reports, including how many reviewers collected data from each report, whether they worked independently, any processes for obtaining or confirming data from study investigators, and if applicable, details of automation tools used in the process.	p. 6
Data items	10a	List and define all outcomes for which data were sought. Specify whether all results that were compatible with each outcome domain in each study were sought (e.g. for all measures, time points, analyses), and if not, the methods used to decide which results to collect.	p. 6
	10b	List and define all other variables for which data were sought (e.g. participant and intervention characteristics, funding sources). Describe any assumptions made about any missing or unclear information.	p. 6
Study risk of bias assessment	11	Specify the methods used to assess risk of bias in the included studies, including details of the tool(s) used, how many reviewers assessed each study and whether they worked independently, and if applicable, details of automation tools used in the process.	p. 6
Effect measures	12	Specify for each outcome the effect measure(s) (e.g. risk ratio, mean difference) used in the synthesis or presentation of results.	p. 6
Synthesis methods	13a	Describe the processes used to decide which studies were eligible for each synthesis (e.g. tabulating the study intervention characteristics and comparing against the planned groups for each synthesis (item #5)).	p. 6
	13b	Describe any methods required to prepare the data for presentation or synthesis, such as handling of missing summary statistics, or data conversions.	p. 6
	13c	Describe any methods used to tabulate or visually display results of individual studies and syntheses.	p. 6
	13d	Describe any methods used to synthesize results and provide a rationale for the choice(s). If meta-analysis was performed, describe the model(s), method(s) to identify the presence and extent of statistical heterogeneity, and software package(s) used.	p. 6
	13e	Describe any methods used to explore possible causes of heterogeneity among study results (e.g. subgroup analysis, meta-regression).	p. 6
	13f	Describe any sensitivity analyses conducted to assess robustness of the synthesized results.	p. 6
Reporting bias assessment	14	Describe any methods used to assess risk of bias due to missing results in a synthesis (arising from reporting biases).	p. 6
Certainty assessment	15	Describe any methods used to assess certainty (or confidence) in the body of evidence for an outcome.	p. 6

Table S1. PRISMA 2020 Checklist

Section & Topic	Item #	Checklist item	Location where item is reported
**RESULTS**			
Study selection	16a	Describe the results of the search and selection process, from the number of records identified in the search to the number of studies included in the review, ideally using a flow diagram.	Figure 1
	16b	Cite studies that might appear to meet the inclusion criteria, but which were excluded, and explain why they were excluded.	Figure 1
Study characteristics	17	Cite each included study and present its characteristics.	Table 1
Risk of bias in studies	18	Present assessments of risk of bias for each included study.	Table 1
Results of individual studies	19	For all outcomes, present, for each study: (a) summary statistics for each group (where appropriate) and (b) an effect estimate and its precision (e.g. confidence/credible interval), ideally using structured tables or plots.	Figure 2
Results of syntheses	20a	For each synthesis, briefly summarise the characteristics and risk of bias among contributing studies.	p. 7
	20b	Present results of all statistical syntheses conducted. If meta-analysis was done, present for each the summary estimate and its precision (e.g. confidence/credible interval) and measures of statistical heterogeneity. If comparing groups, describe the direction of the effect.	p. 10 & 11
	20c	Present results of all investigations of possible causes of heterogeneity among study results.	p. 10 & 11
	20d	Present results of all sensitivity analyses conducted to assess the robustness of the synthesized results.	p. 10 & 11
Reporting biases	21	Present assessments of risk of bias due to missing results (arising from reporting biases) for each synthesis assessed.	n/a
Certainty of evidence	22	Present assessments of certainty (or confidence) in the body of evidence for each outcome assessed.	p. 10
**DISCUSSION**			
Discussion	23a	Provide a general interpretation of the results in the context of other evidence.	p. 11 & 12
	23b	Discuss any limitations of the evidence included in the review.	p. 13 & 14
	23c	Discuss any limitations of the review processes used.	p. 13 & 14
	23d	Discuss implications of the results for practice, policy, and future research.	p. 13 & 14
**OTHER INFORMATION**			
Registration and protocol	24a	Provide registration information for the review, including register name and registration number, or state that the review was not registered.	p. 5
	24b	Indicate where the review protocol can be accessed, or state that a protocol was not prepared.	p. 5
	24c	Describe and explain any amendments to information provided at registration or in the protocol.	n/a
Support	25	Describe sources of financial or non-financial support for the review, and the role of the funders or sponsors in the review.	p. 14
Competing interests	26	Declare any competing interests of review authors.	p. 14
Availability of data, code and other materials	27	Report which of the following are publicly available and where they can be found: template data collection forms; data extracted from included studies; data used for all analyses; analytic code; any other materials used in the review.	p. 14

From: Page MJ, McKenzie JE, Bossuyt PM, Boutron I, Hoffmann TC, Mulrow CD, et al. The PRISMA 2020 statement: an updated guideline for reporting systematic reviews. BMJ 2021;372:n71. doi: 10.1136/bmj.n71. This work is licensed under CC BY 4.0. To view a copy of this license, visit https://creativecommons.org/licenses/by/4.0/

Appendix S1. Search Strategy from Selected Databases

Scopus

Advanced query

((tuberculosis MeSH Terms) AND ("severe acute malnutrition" MeSH Terms)) OR ((("severe acute malnutrition" Text Word) OR (kwashiorkor Text Word) OR ("protein-energy malnutrition" Text Word) OR (marasmus Text Word) OR ("severe wasting" Text Word) OR ("nutritional oedema" Text Word) OR ("nutritional edema" Text Word)) AND (tuberculosis Text Word))

Show less

Edit in advanced search

Documents | Preprints (Beta) | Secondary documents

Are you searching for: ((tuberculosis[MeSH Terms]) AND ("severe acute malnutrition"[MeSH Terms])) OR ((...

21 documents found

PubMed

History and Search Details | Download | Delete

Search	Actions	Details	Query	Results	Time
#1	•••	! >	Search: **((tuberculosis[MeSH Terms]) AND ("severe acute malnutrition"[MeSH Terms])) OR ((((("severe acute malnutrition"[Text Word]) OR (kwashiorkor[Text Word])) OR ("protein-energy malnutrition"[Text Word])) OR (marasmus[Text Word])) OR ("severe wasting"[Text Word])) OR ("nutritional oedema"[Text Word])) OR ("nutritional edema"[Text Word])) AND (tuberculosis[Text Word]))** Sort by: **Most Recent**	257	07:43:35

Web of Science

102 results from Web of Science Core Collection for:

(TS=(severe acute malnutrition)) AND TS=(tuberculosis)

Copy query link

+ Add Keywords | Quick add keywords: | + severe acute malnutrition | + survival status | + severe malnutrition | + retrospective cohort | + wast

102 Documents | You may also like... | Analyze Results | Citation Report | Create Alert

Embase

Enter search query e.g. 'cancer gene therapy'/exp OR ((treatment OR therapy) NEAR/5 fluorouracil):ab

('tuberculosis'/exp AND 'severe acute malnutrition'/exp OR (('severe acute malnutrition' OR kwashiorkor OR 'protein-energy malnutri

Mapping | Date | Fields | Quick limits

Search history

Select all | Combine | using AND | Save | Delete | Export | Email

#1	('tuberculosis'/exp AND 'severe acute malnutrition'/exp OR (('severe acute malnutrition' OR kwashiorkor OR 'protein-energy malnutrition' OR marasmus OR 'severe wasting') AND tuberculosis)) AND 'severe acute malnutrition' AND 'tuberculosis'	172	⋮

172 results for search #1 | Create email alert | Index miner

APPENDIX S2. RISK-OF-BIAS AND STUDY-QUALITY ASSESSMENTS

Cohort studies

The adapted Newcastle-Ottawa Scale contained six assessment items. Points were additive, and criteria that were not met or not reported received 0 points. Possible total scores ranged from 0 to 9.

Assessment criteria and scoring

No.	Assessment item	Scoring rule	Max.
**1**	**Representativeness of the population at risk**	**1 point:** Children with SAM were recruited from a representative setting (multicenter, community, outpatient, or inpatient hospital).<br>**1 point:** Systematic sampling was clearly described (e.g., consecutive sampling or interval sampling of every X children).	**2**
**2**	**Ascertainment of SAM status**	**1 point:** The SAM definition was clearly stated in the article.<br>**0 points:** SAM was not clearly defined.	**1**
**3**	**Outcome presence (TB diagnosis timing)**	**1 point:** TB was diagnosed at the time of SAM diagnosis or prospectively from that point, with no pre-existing TB.<br>**0 points:** Timing was not clearly described or pre-existing TB was possible.	**1**
**4**	**Study comparability: factors available for subgroup analyses**	**0 points:** No subgroup factors were available.<br>**1 point:** One factor was available.<br>**2 points:** Two or more factors were available.<br>**Examples:** age, HIV status, TB contact, BCG status, sex, setting, and season.	**2**
**5**	**Assessment of the TB outcome**	**1 point:** Any detail was provided about how participants were assessed for TB.<br>**1 point:** TB was confirmed using microbiology and/or standardized clinical criteria (e.g., Graham et al., 2015).	**2**
**6**	**Follow-up duration**	**1 point:** Follow-up lasted 2 months or longer to confirm the TB diagnosis.<br>**0 points:** Follow-up lasted less than 2 months or was unclear.	**1**

Interpretation of the total score (maximum = 9)

Total score	Methodological quality	Corresponding risk of bias
**7-9**	High	Low
**4-6**	Moderate	Moderate
**0-3**	Low	High

<u>**Cross-sectional studies**</u>

The adapted Joanna Briggs Institute assessment contained eight items. For every item, Yes = 1 and No = 0, yielding possible total scores from 0 to 8.

Assessment criteria and scoring

No.	Assessment item	Max.
**1**	Were the criteria for inclusion in the sample clearly defined?	**1**
**2**	Were the study participants and setting described in detail?	**1**
**3**	Was SAM clearly defined?	**1**
**4**	Was it clear that children with SAM were systematically sampled (e.g., consecutive sampling or interval sampling of every X children)?	**1**
**5**	Was any detail provided about how study participants were assessed for TB?	**1**
**6**	Were TB case definitions based on microbiologic and/or standardized clinical criteria (e.g., Graham et al.)?	**1**
**7**	Was at least one factor available for subgroup analyses (e.g., age, HIV status, TB contact, BCG status, sex, setting, or season)?	**1**
**8**	Was more than one factor available for subgroup analyses (e.g., age, HIV status, TB contact, BCG status, sex, setting, or season)?	**1**

Interpretation of the total score (maximum = 8)

Total score	Methodological quality	Corresponding risk of bias
**6-8**	High	Low
**3-5**	Moderate	Moderate
**0-2**	Low	High

Abbreviations. BCG, Bacillus Calmette-Guerin;; SAM, severe acute malnutrition; TB, tuberculosis.

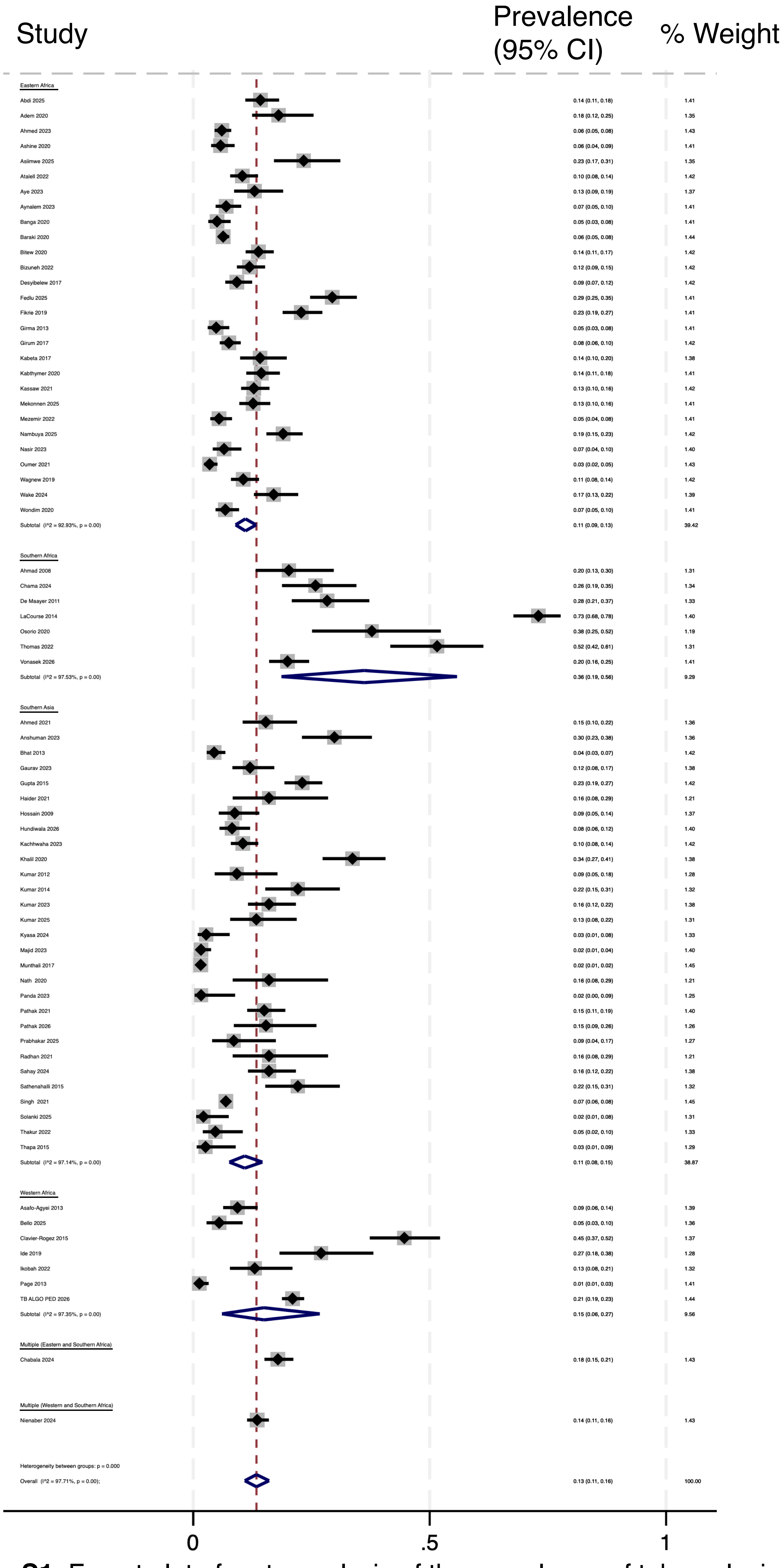

Figure S1. Forest plot of meta-analysis of the prevalence of tuberculosis in children with severe acute malnutrition with studies stratified by United Nations geoscheme subregion. CI: confidence interval.

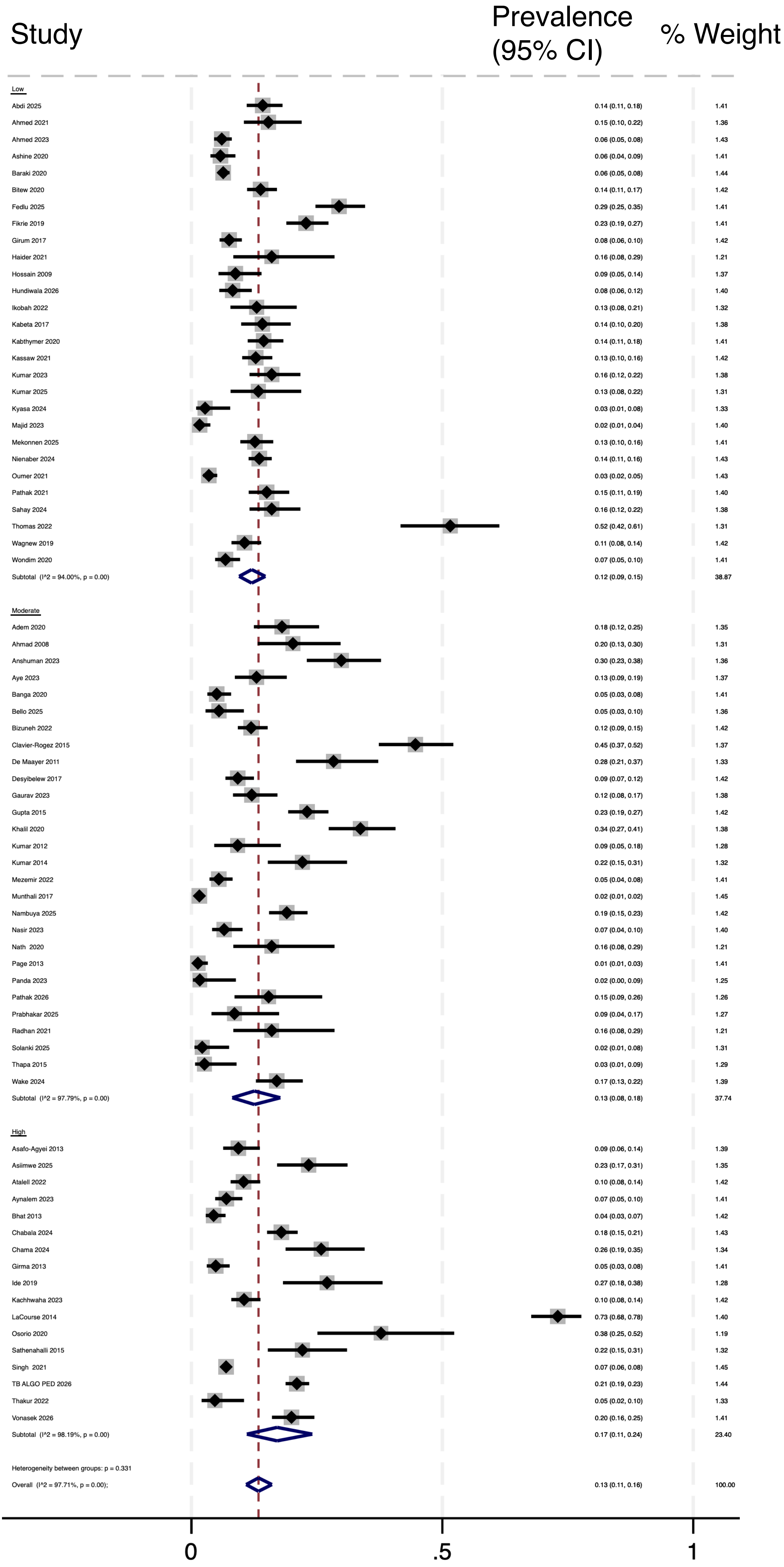

Figure S2. Forest plot of meta-analysis of the prevalence of tuberculosis in children with severe acute malnutrition with studies stratified by quality. CI: confidence interval.

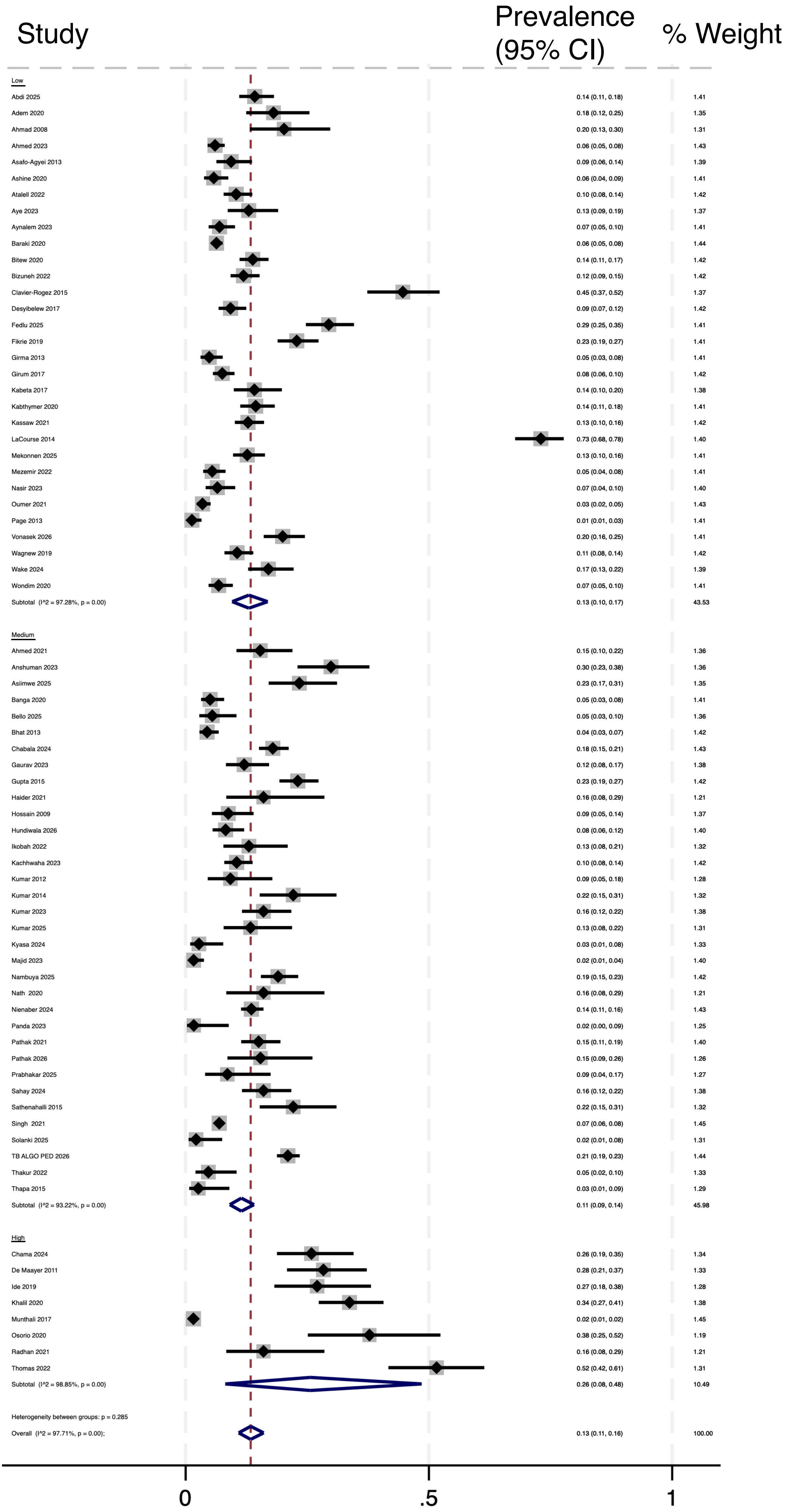

Figure S3. Forest plot of meta-analysis of the prevalence of tuberculosis in children with severe acute malnutrition with studies stratified by national tuberculosis annual incidence (low: <150 per 100,000; medium: 150 to 250 per 100,000; high: >250 per 100,000). CI: confidence interval.

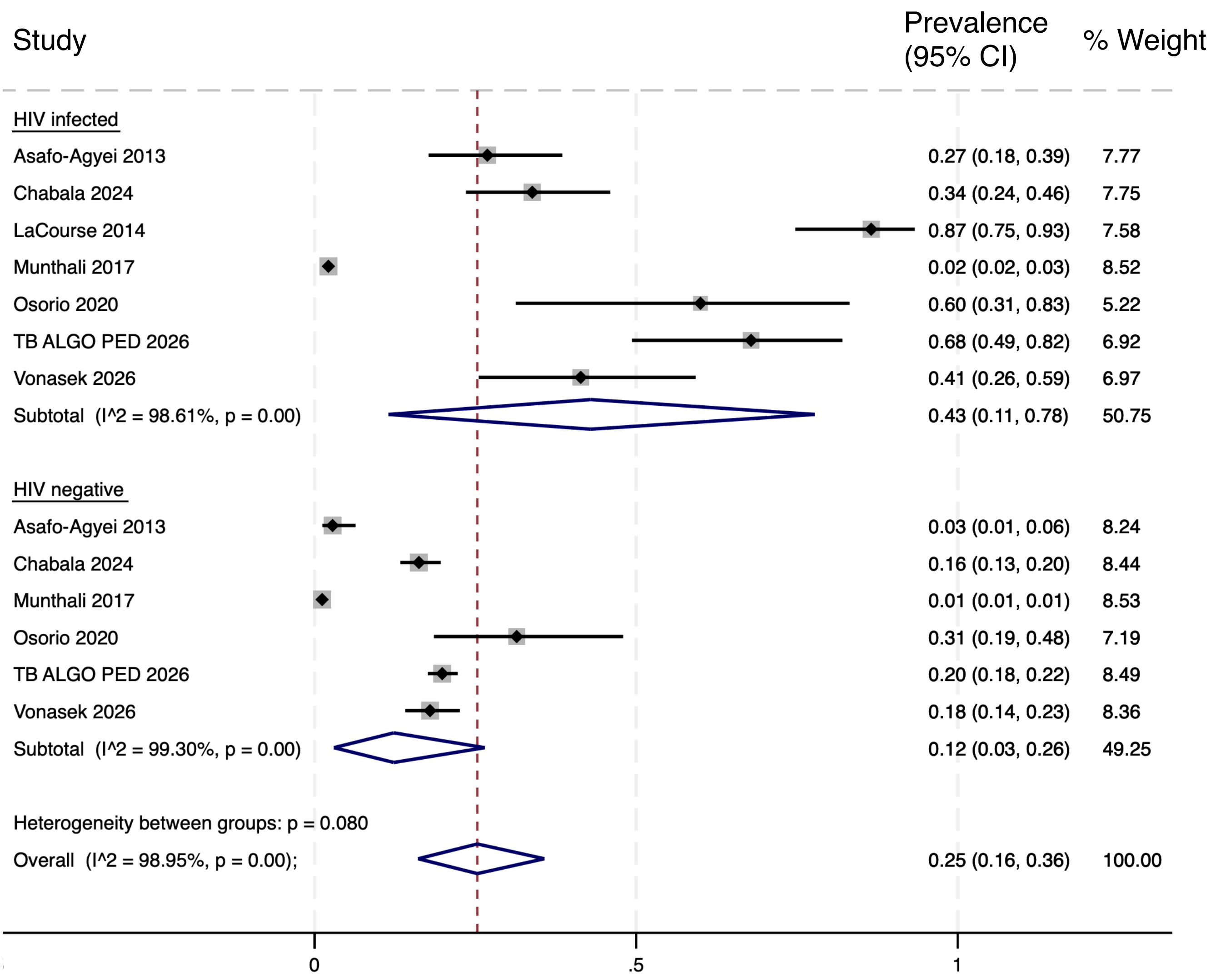

Figure S4. Forest plot of meta-analysis of the prevalence of tuberculosis in children with severe acute malnutrition—sub-group analysis by HIV status. CI: confidence interval.

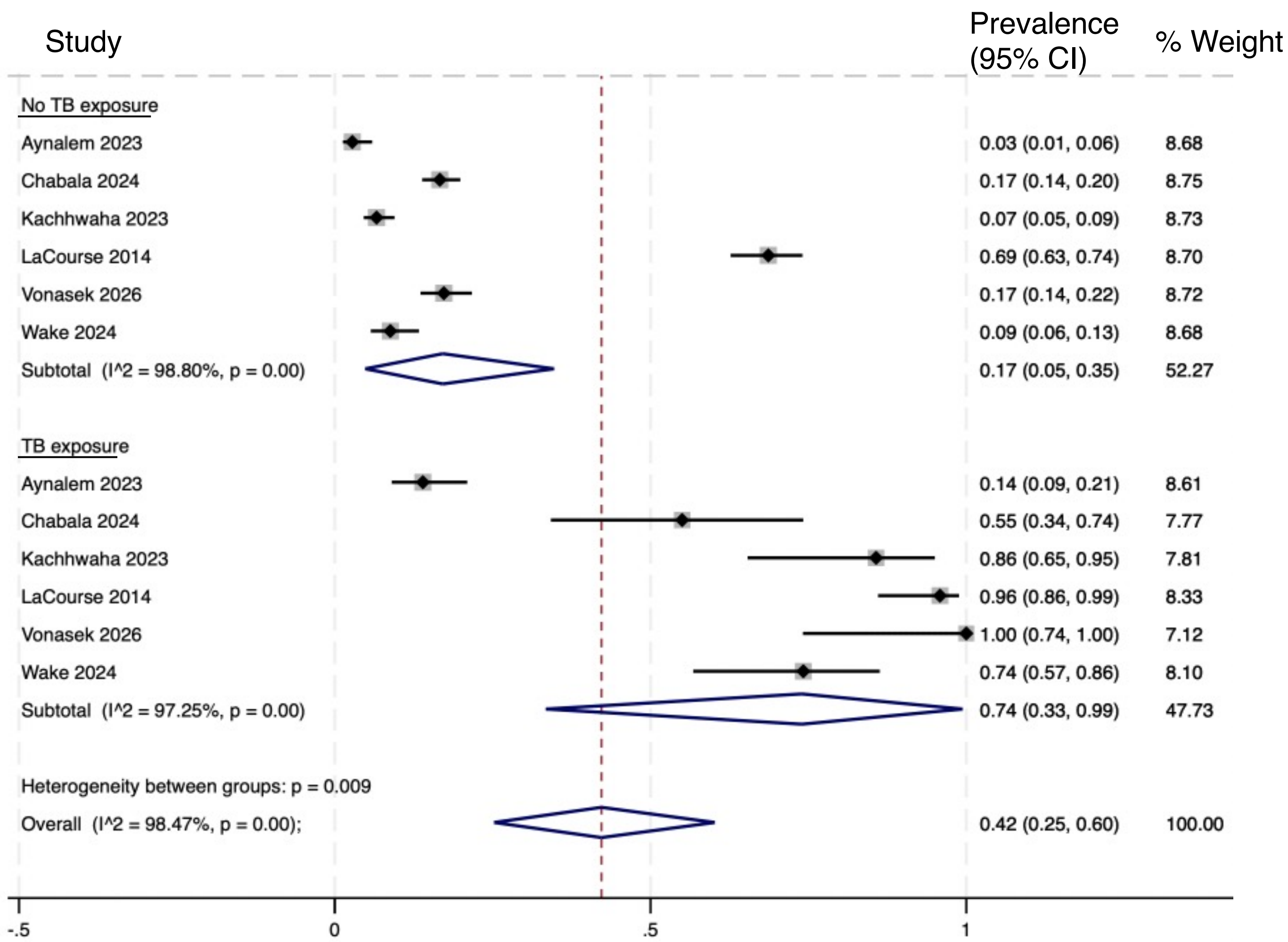

Figure S5. Forest plot of meta-analysis of the prevalence of tuberculosis in children with severe acute malnutrition—sub-group analysis by presence or absence of history of exposure to an individual with tuberculosis. CI: confidence interval.

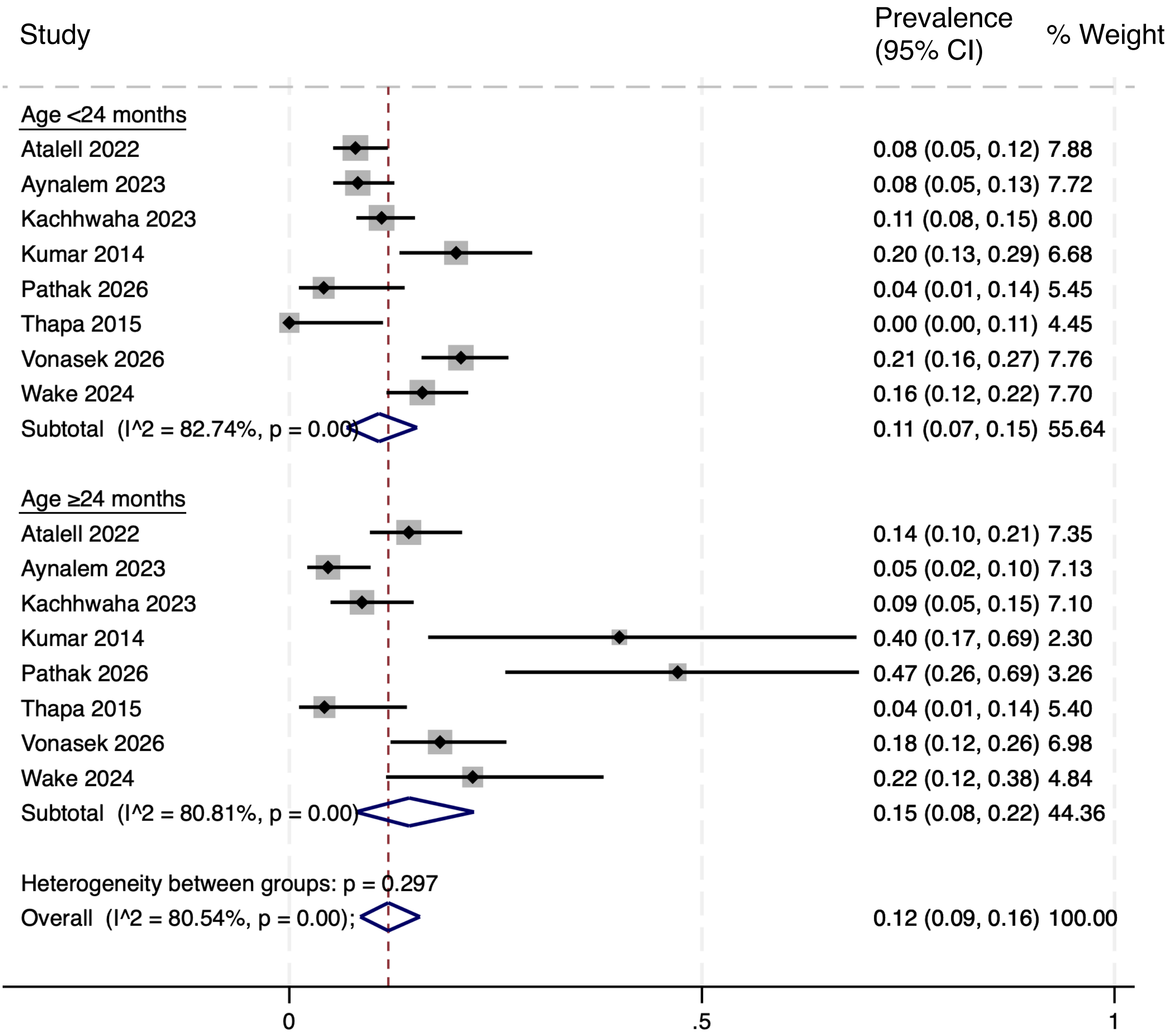

Figure S6. Forest plot of meta-analysis of the prevalence of tuberculosis in children with severe acute malnutrition—sub-group analysis by age. CI: confidence interval.

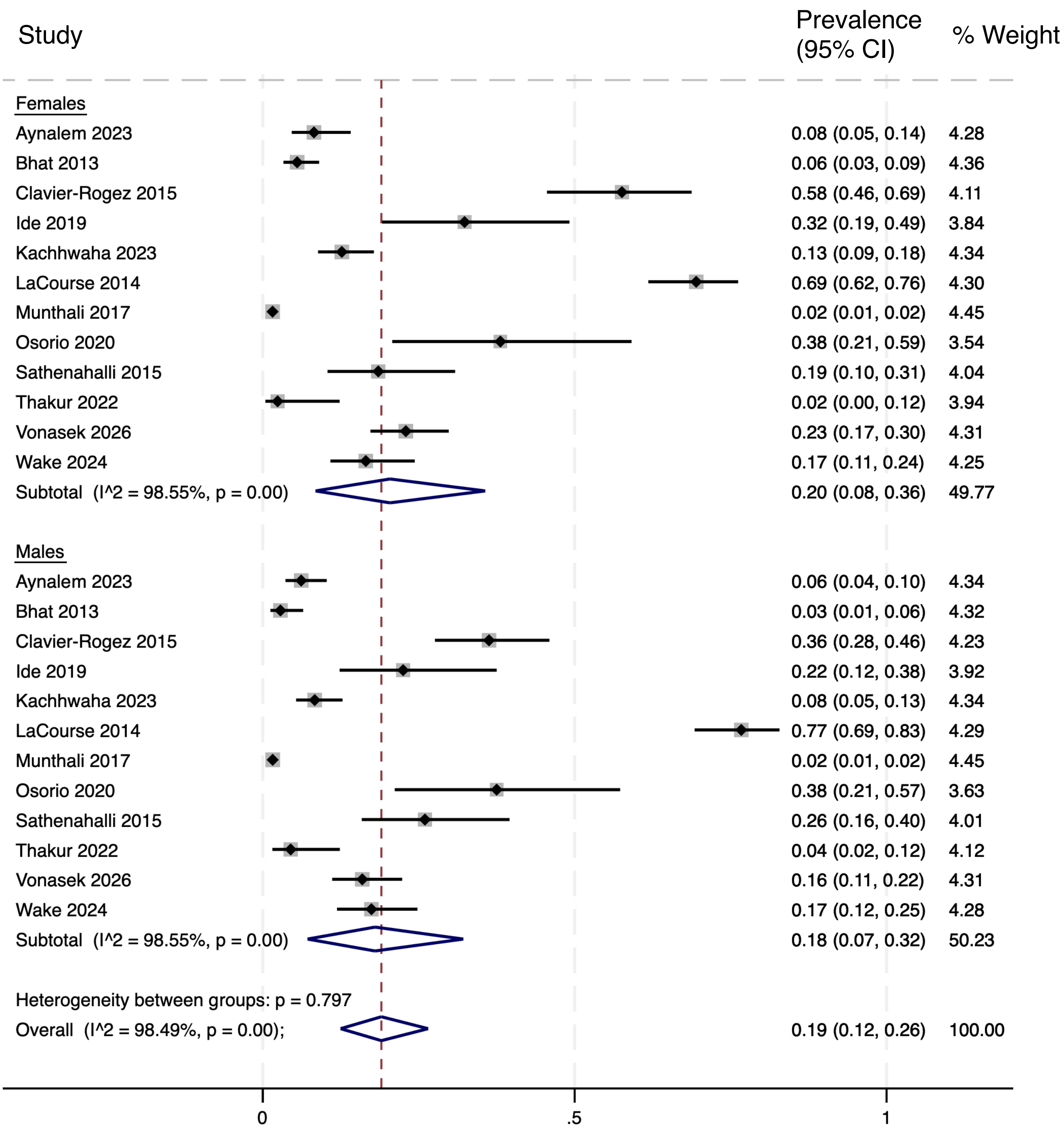

Figure S7. Forest plot of meta-analysis of the prevalence of tuberculosis in children with severe acute malnutrition—sub-group analysis by sex. CI: confidence interval.

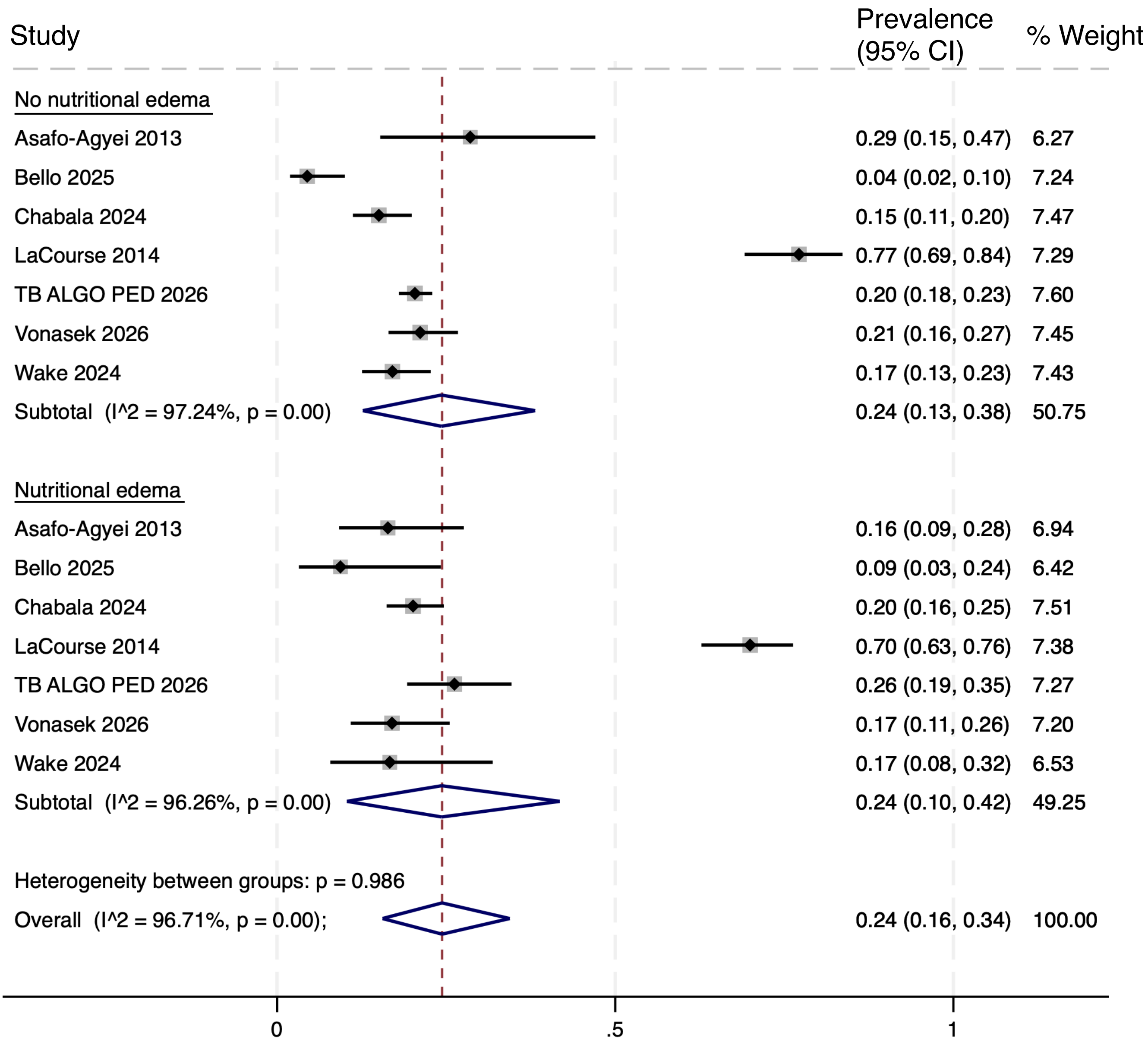

Figure S8. Forest plot of meta-analysis of the prevalence of tuberculosis in children with severe acute malnutrition—sub-group analysis by presence or absence of nutritional edema. CI: confidence interval.

Table S2. Sensitivity analysis of pooled prevalence of TB among children with SAM by stratified and subgroup meta-analyses with a single study outlier (LaCourse 2014) included and excluded.

	**Outlier Study Included** *(same as Table 2)*				**Outlier Study Excluded**			
	**N**	**Prevalence (95% CI)**	**I^2**	***p*-value**	**N**	**Prevalence (95% CI)**	**I^2**	***p*-value**
All studies	73	13% (11-16%)	98%	-	72	13% (11-15%)	97%	-
<u>UN geoscheme subregion</u>								
Eastern Africa	28	11% (9-13%)	93%		28	11% (9-13%)	93%	
Southern Africa	7	36% (19-56%)	98%	<0.001	6	30% (21-40%)	87%	<0.001
Southern Asia	29	11% (8-15%)	97%		29	11% (8-15%)	97%	
Western Africa	7	15% (6-27%)	97%		7	15% (6-27%)	97%	
<u>Study quality</u>								
Low	28	12% (9-15%)	94%	0.331	28	12% (9-15%)	94%	0.702
Moderate	28	13% (8-18%)	98%		28	13% (8-18%)	98%	
High	17	17% (11-24%)	98%		16	14% (10-18%)	96%	
<u>Nation annual TB incidence</u>								
Low (<150 per 100,000)	31	13% (10-17%)	97%	0.285	30	11% (9-14%)	94%	0.331
Medium (150-250 per 100,000)	34	11% (9-14%)	93%		34	11% (9-14%)	93%	
High (>250 per 100,000)	8	26% (8-48%)	99%		8	26% (8-48%)	99%	
<u>HIV status</u>								
Infected	7	43% (11-78%)	99%	0.080	6	35% (9-66%)	98%	0.136
Negative	6	12% (3-26%)	99%		6	12% (3-26%)	99%	
<u>History of TB exposure</u>								
None	6	17% (5-35%)	99%	0.009	5	10% (5-16%)	93%	0.003
Present	6	74% (33-99%)	97%		5	68% (27-98%)	96%	
<u>Age</u>								
<24 months	8	11% (7-15%)	83%	0.297	-	-	-	-
≥24 months	8	15% (8-22%)	81%					
<u>Sex</u>								
Female	12	20% (8-36%)	99%	0.797	11	16% (8-27%)	97%	0.633
Male	12	18% (7-32%)	99%		11	13% (6-22%)	97%	
<u>SAM type</u>								
No nutritional edema	7	24% (13-38%)	97%	0.986	6	16% (12-22%)	83%	0.349
Nutritional edema present	7	24% (10-42%)	96%		6	19% (16-23%)	19%	

CI: confidence interval, N: number of studies in the meta-analysis, SAM: severe acute malnutrition, TB: tuberculosis, UN: United Nations
